# Distinct epigenetic modulation of differentially expressed genes in the adult mouse brain following prenatal exposure to low-dose bisphenol A

**DOI:** 10.1007/s10565-024-09875-4

**Published:** 2024-05-22

**Authors:** Jie Weng, Yue-yan Zhu, Li-yong Liao, Xin-tong Yang, Yu-hao Dong, Wei-da Meng, Dai-jing Sun, Yun Liu, Wen-zhu Peng, Yan Jiang

**Affiliations:** 1 Institutes of Brain Science, State Key Laboratory of Medical Neurobiology and MOE Frontiers Center for Brain Science, Fudan University, Shanghai, 200032 China; 2 Shanghai Medical college, Fudan University, Shanghai, 200032 China; 3 The MOE Key Laboratory of Metabolism and Molecular Medicine, Department of Biochemistry and Molecular Biology, School of Basic Medical Sciences, Fudan University, Shanghai, 200032 China

**Keywords:** Bisphenol A, Maternal stress, Transcriptomic profiling, Epigenetic regulation, Sex-specific

## Abstract

Bisphenol A (BPA) is a common component in the manufacture of daily plastic consumer goods. Recent studies have suggested that prenatal exposure to BPA can increase the susceptibility of offspring to mental illness, although the underlying mechanisms remain unclear. In this study, we performed transcriptomic and epigenomic profiling in the adult mouse brain following prenatal exposure to low-dose BPA. We observed a sex-specific transcriptional dysregulation in the cortex, with more significant differentially expressed genes was observed in adult cortex from male offspring. Moreover, the upregulated genes primarily influenced neuronal functions, while the downregulated genes were significantly associated with energy metabolism pathways. More evidence supporting impaired mitochondrial function included a decreased ATP level and a reduced number of mitochondria in the cortical neuron of the BPA group. We further investigated the higher-order chromatin regulatory patterns of DEGs by incorporating published Hi-C data. Interestingly, we found that upregulated genes exhibited more distal interactions with multiple enhancers, while downregulated genes displayed relatively short-range interactions among adjacent genes. Our data further revealed decreased H3K9me3 signal on the distal enhancers of upregulated genes, whereas increased DNA methylation and H3K27me3 signals on the promoters of downregulated genes. In summary, our study provides compelling evidence for the potential health risks associated with prenatal exposure to BPA, and uncovers sex-specific transcriptional changes with a complex interplay of multiple epigenetic mechanisms.

**Graphical abstract:**

1. Distinct chromatin interaction pattern of DEGs in the cortex of adult male offspring in response to prenatal BPA exposure.

2. Upregulated genes exhibited intensive and long-range chromatin interactions, with decreased H3K9me3 modification on the distal enhancers.

3. Downregulated genes were featured by promoter-promoter interactions among adjacent genes and increased DNA methylation and H3K27me3 modification at the promoter regions.

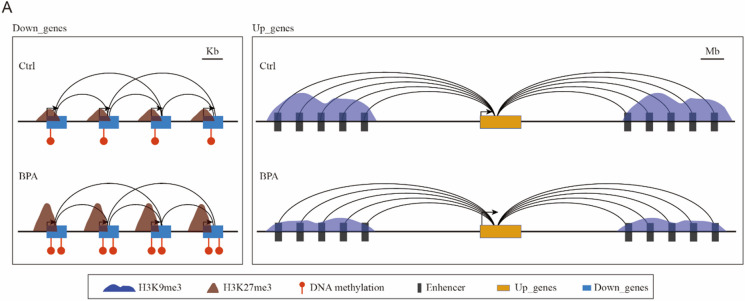

**Supplementary information:**

The online version contains supplementary material available at 10.1007/s10565-024-09875-4.

## Introduction

Bisphenol A (BPA) is extensively used in the manufacture of daily consumers. According to a report on the “Research and Markets”, global consumption of BPA reached 7.7 million tons in 2015, and the annual demand continues to rise. BPA can leach from products under certain conditions and enter the human body through multiple pathways (Chen et al. 2016). Studies have shown that BPA exposure is common worldwide (Vandenberg et al. 2010). In the United States from 2003 to 2004, 96% of surveyed women had detectable levels of BPA in their urine (Hoyt et al. 2011). Health Canada reported that BPA was detected in the urine of 95% of the population (Health Canada 2013). Therefore, the safety for BPA remains a potential concern for global health. Previous studies have documented the complex effects of BPA on human health, underscoring the perinatal period as the most vulnerable window of exposure. BPA accumulates in the embryo due to the absence of active metabolic enzymes in the fetal liver (Jalal et al. 2018) and can be detected in the plasma, serum, and placenta of newborns (Vandenberg et al. 2010). Epidemiological studies have shown a significant correlation between perinatal BPA exposure and elevated behavioral abnormality in adolescents (Ejaredar et al. 2017), and such correlation was also reported in mouse models (Wolstenholme et al. 2013; Xu et al. 2012).

BPA has potential influence on brain function, primarily through its mimicry of estrogen, a hormone critical for brain development. It has been reported to impair neurogenesis and synaptogenesis, and disrupt neurotransmission in various brain regions, including hypothalamus (Arambula et al. 2016), hippocampus (Thongkorn et al. 2023), and prefrontal cortex (Kanlayaprasit et al. 2021). Moreover, prenatal BPA exposure has been associated with sex-specific disruptions in the transcriptome of the neonatal amygdala, along with impairments of the estrogen, oxytocin, and vasopressin signaling pathways (Arambula et al. 2018). Epigenetic mechanisms mediate lasting alterations in gene expression without the alterations in the DNA sequence, and DNA methylation and histone modifications are two major types of contributors. DNA methylation has been primarily studied in the context of BPA’s effect on brain functions (Besaratinia 2023). Prenatal BPA exposure has been shown to alter global DNA methylation in the fetal mouse brain (Yaoi et al. 2008). Additional studies have demonstrated changes of DNA methylation on developing genes, including estrogen receptor 1/2 (*Esr1/2*) (Kundakovic et al. 2013), potassium-chloride cotransporter 2 (*Kcc2*) (Yeo et al. 2013), and NMDA receptor subunit 2b (*Grin2b*) (Alavian-Ghavanini et al. 2018). Histone modifications have also been implicated in regulating BPA's actions in the brain. Decreased histone modifications, including H3K9ac, H4K8ac, and H3K9me3 has been detected upon BPA exposure (Jiang et al. 2016; Senyildiz et al. 2017).

In this study, we first conducted RNA-seq on the prefrontal cortex of adult mouse with prenatal exposure to a low-dose of BPA. We then integrated neuronal 3D genome architecture analysis to assess higher-order chromatin folding patterns associated with the identified differentially expressed genes (DEGs). Additionally, we conducted epigenomic profiling for DNA methylation, H3K27me3, and H3K9me3 to examine their roles in DEGs. Our study revealed the complex epigenetic regulations of gene expression in the adult brain following prenatal BPA exposure, shedding light on its potential effects on brain function.

## Results

### Behavioral assessment of adult offspring after prenatal exposure to BPA

Timed-pregnant females were randomly assigned to the control (Ctrl) and BPA groups. Corn oil or BPA with a dosage of 40 µg/kg bw/day (body weight per day) was administered via oral gavage from gestational day (GD) 0.5 to GD 13.5, respectively (Fig. S1A). We collected 12–14 litters from each group and found no significant difference in sex ratio between the Ctrl and BPA groups (Fig. S1B). We then evaluated behavioral performance of adult offspring in multiple paradigms, including open field, elevated plus maze, tail suspension, forced swim. Only mild abnormality, if any, was observed in the BPA compared to the Ctrl group at this low dosage of exposure (Fig. S1C-D). Given the widely reported sex-effects in BPA studies (Rebuli and Patisaul 2016), we analyzed data from both sexes separately. In the male offspring, we observed a significant increase in total distance in open field test (n = 26–29/group), but no significant change was detected in other paradigms (Fig. S1C). No significant behavioral changes were observed in females except increased time in the center from open filed test (n = 15–30/group) (Fig. S1D). We then collected brains at the age of 4–5-month-old and performed transcriptomic (RNA-seq) and epigenomic profiling for DNA methylation (Reduced Representation Bisulfite Sequencing, RRBS), H3K27me3 and H3K9me3 (ChIP-seq) (Fig. S1A). For the molecular study, we selected animals from different litters to minimize any potential litter effect.

#### Downregulation of genes in energy metabolic pathway in adult cortex after prenatal BPA exposure

**Fig. 1:**
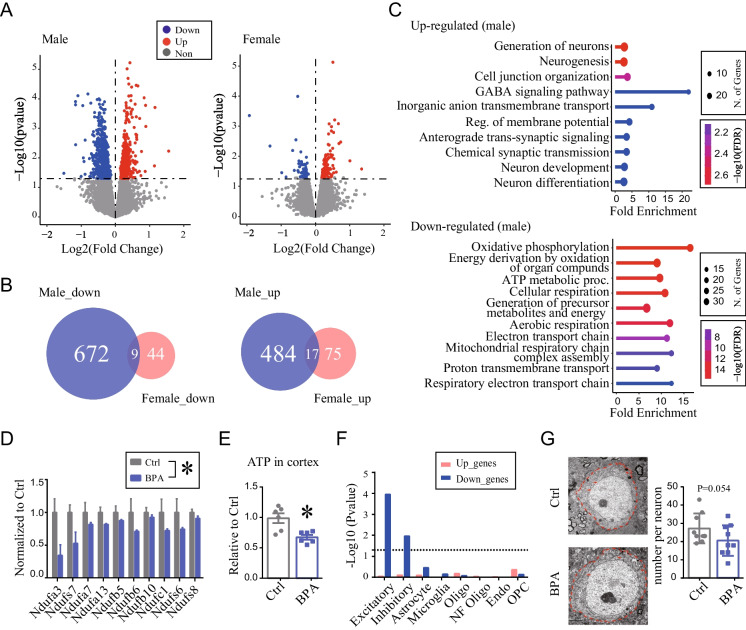
Impaired mitochondrial function in adult cortex after prenatal low-dose BPA exposure. **(A)** Volcano plot of DEGs in adult prefrontal cortex of male offspring and female offspring. Red, up-regulated genes; blue, down-regulated genes. BPA vs. Ctrl, *P* < 0.05, n = 3/group. **(B)** Overlapped down-regulated genes (Left) and up-regulated genes (Right) between male and female. **(C)** ShinyGO analysis (“Biological Process”) of up- (Top) and down-regulated (Bottom) genes (*P* < 0.05) of male offspring. **(D)** qRT-PCR validation in male cortex, n = 6/group. Mean ± SEM. Two-way ANOVA. BPA effect, *P < 0.05 **(E)** ATP levels in the cortex of adult male offspring measured by ATP Detection Assay Kit. n = 6/group. Mean ± SEM. *P < 0.05, Unpaired t test, two-tailed. **(F)** Bar plots show the gene set enrichment score (-log_10_*P*value) of “Down_genes” and “Up_genes” in each cell types. The eight cell-types specific marker genes were identified using the published scRNA-seq (GSE124952). Notably, only “Down_genes” was significantly enriched in neurons. The dashed line represents the significance cutoff threshold at *P* = 0.05. (**G)** Transmission electron microscopy (TEM) image of prefrontal cortex in adult male offspring. (Left) Neurons are depicted by red dashed line. Red arrows indicate mitochondria. Scale bar, 2 μm. (Right) Statistical analysis of the number of mitochondria in individual neuron. N = 9/group. Mean ± SEM. *P* = 0.054, unpaired *t* test, one-tailed

RNA-seq was performed on the prefrontal cortex of adult offspring from both sexes (Fig. 1). Despite the mild behavioral changes observed in male offspring (Fig. S1C), significant transcriptional changes were observed in response to prenatal BPA exposure, with 1,182 significant differentially expressed genes (DEGs) identified between the BPA and Ctrl groups (*P* < 0.05, n = 3/group) (Fig. 1A, left, Table S1). However, the impact of prenatal BPA exposure on females was considerably less pronounced, with only 145 genes showing significant changes (*P* < 0.05, n = 3/group) (Fig. 1A, right, Table S2). Furthermore, there was minimal overlap in DEGs between males and females, with only 9 upregulated and 17 downregulated genes common to both (Fig. 1B). These results, together with findings from the literatures (Farabollini et al. 2002; Harley et al. 2013), suggest that males are more vulnerable to prenatal BPA exposure. This also corroborates reported sex differences in response to adverse environmental events during brain development (Pérez-Cerezales et al. 2018).

We focused on males for subsequent analysis. Gene Ontology (GO) analysis of the male DEGs revealed that upregulated genes were enriched in the pathways associated with neuronal functions, including neurogenesis, neuron differentiation and chemical synaptic transmission (Fig. 1C, top). Meanwhile, downregulated genes were primarily enriched in the oxidative phosphorylation (OXPHOS) pathway and other energy metabolic pathways (Fig. 1C, bottom). This finding was also confirmed by Gene Set Enrichment Analysis (GSEA), which assesses the general trend of all detected genes without applying a cutoff threshold for DEGs. The OXPHOS pathway was at the top of the list of significantly downregulated pathways associated with the BPA group (FDR < 0.05) (Fig. S2). Furthermore, we performed qRT-PCR and validated the decreased expression of genes in mitochondrial Complex I in the BPA group, compared to the Ctrl group, using different batch of samples (*P* < 0.05, n = 6/group) (Fig. 1D).

In addition, we performed an integrated analysis of our bulk RNA-seq, referencing the published scRNA-seq data (Bhattacherjee et al. 2019). When visualizing the average expression of DEGs on the t-SNE plots of the published scRNA-seq, both upregulated (Fig. S3B, D) and downregulated (Fig. S3C, E) genes exhibited relatively high expression in excitatory neurons. However, no significant enrichment was calculated for the upregulated DEGs, while the downregulated DEGs were significantly enriched in both excitatory and inhibitory neurons (Fig. 1F). We performed additional analysis using a different set of published scRNA-seq data (GSE211099). Despite some variations, we consistently observed enrichment of downregulated genes in excitatory neurons (Fig. S4). Given the robust nature of energy metabolism in neurons, and the observed downregulation of genes associated to energy metabolic pathway (Fig. 1C-D, Fig. S2), we speculated that prenatal BPA exposure may potentially influence the mitochondrial function of neurons in adult offspring. To explore this further, we measured ATP levels and observed a significant decrease in the cortex of the BPA compared to the Ctrl group (*P* < 0.05, n = 6/group) (Fig. 1E). Additionally, we examined mitochondrial morphology under electron microscopy. We observed a trend (*P* = 0.054, n = 9/group) towards fewer mitochondria in neurons in the BPA group (Fig. 1G). Collectively, these findings suggested potentially compromised energy metabolism in the adult brain following prenatal exposure to BPA.

#### Differential regulation patterns of DEGs via chromatin interactions in adult cortex after prenatal BPA exposure

**Fig. 2:**
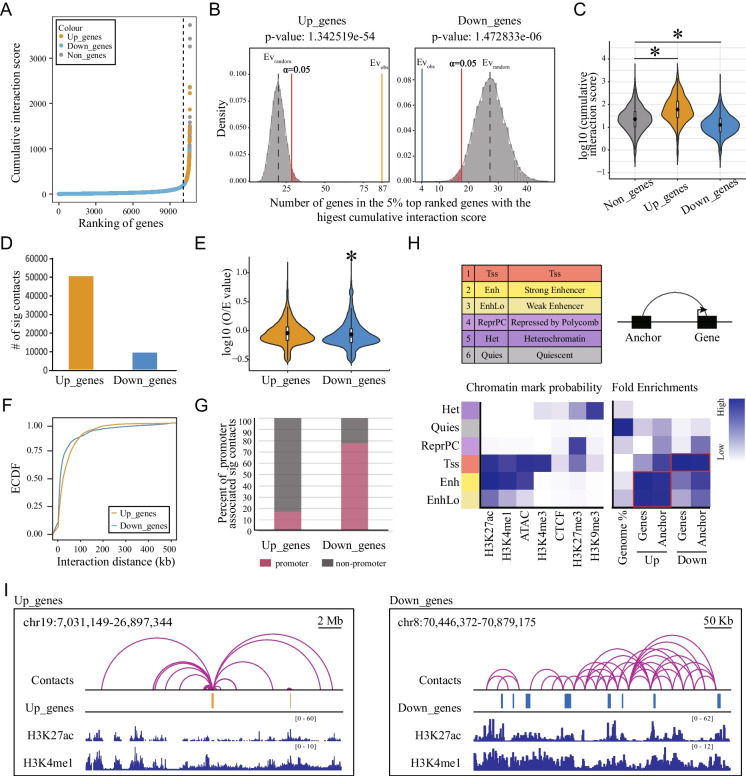
Distinct 3D epigenomic signature on the upregulated and downregulated genes. Published Hi-C data (GSE99363) were used to obtain gene-associated contacts. **(A)** Gene-associated contacts were extracted out and the cumulative interaction scores for each gene were calculated. Genes were ranked according to the cumulative interaction scores. The dashed line separates the 5% top genes with highest cumulative interaction score with other genes. **(B)** Permutation plot of number of overlapped genes between the randomly sampled background genes and the 5% top ranked genes. Yellow and blue line indicates the overlap observed between the up- (Left) or down-regulated (Right) genes and the 5% top ranked genes. **(C)** Violin plot shows distribution of cumulative interaction score of each gene. *P < 0.05, Dunn's test. **(D)** Bar plot shows the number of significant contacts associated with up- and down-regulated genes. Gene-associated significant contacts were defined as either anchor of the contacts overlapping with genes. **(E)** Violin plot shows the distribution of O/E value of each contact. **(F)** ECDF plot of interaction distance shows more proportion of long-range interactions for Up_genes. **(G)** Bar plot shows the proportion of promoter associated with significant contacts. **(H)** Chromatin states were categorized by ChromHMM using our ATAC-seq, H3K27me3, H3K9me3 ChIP-seq data and published H3K27ac (GSE99363), CTCF (GSE99363), H3K4me1 (GSE90020), H3K4me3 (GSE90020) ChIP-seq datasets. The enrichment score for the categories is shown by color. The red box highlights the most significant enrichment of categories. **(I)** Representative map tracks show chromatin contacts and enhancer marks (H3K27ac, H3K4me1) associated with Up_genes (Left) and Down_genes (Right)

Transcriptomic analysis reveals that prenatal BPA exposure leads to alterations in numerous functional pathways in the adult cortex (Fig. 1). These changes encompass the collective expression of a vast number of genes, suggesting a generalized regulatory mechanism as opposed to one restricted to individual genes. Previous research has shown that chromatin interactions, within the scope of spatial chromatin organization, are essential drivers of the coordinated regulation of multiple genes (Rajarajan et al. 2018). Therefore, we utilized published Hi-C data to gain deep insight into the transcriptional regulation patterns of DEGs in our current study (Jiang et al. 2017). We first reconstruct the chromatin contact map, retrieving significant contacts at a resolution of 20 Kb. We then selected the significant contacts associated with all genes detected in our bulk RNA-seq and computed the cumulative interaction score for each gene. Intriguingly, when we arranged the genes according to their interaction scores, the upregulated genes were significantly overrepresented among the top 5% (Fig. 2A, B left). In contrast, downregulated genes were underrepresented in this population (Fig. 2A, B right). Violin plots depict the distribution of interaction scores for upregulated (Up_genes), downregulated (Down_genes), and unchanged (Non_genes) genes. Consistently, the interaction scores of Up_genes were significantly higher compared to those of Non_genes, while Down-genes exhibited the opposite pattern (Fig. 2C). The number of significant contacts associated with Up_genes was larger compared to Down_genes (Fig. 2D), indicating more interactions with Up_genes. O/E value reflets the intensity for each contact, and the contact intensity for Up_genes was higher compared to Down_genes (Fig. 2E), indicating stronger interactions for Up_genes. Moreover, empirical cumulative distribution function (ECDF) plot demonstrated the distribution of interaction distance, and revealed higher proportion of Up_genes contacts with long distances as compared to Down_genes (Fig. 2F). Collectively, these data indicated that there were more and stronger chromatin interactions with longer distances associated with Up_genes compared to Down_genes. Notably, there was a much higher proportion of interactions associated with promoters for Down_genes (80%), compared with 20% for Up_genes (Fig. 2G). We also performed additional analysis with a different set of published Hi-C data (Chandrasekaran et al. 2021) and obtained consistent results (Fig. S5A-G).

We further applied ChromHMM analysis to assess the chromatin states for both genes and their corresponding anchors of chromatin interactions associated with upregulated and downregulated genes. Using our ChIP-seq (H3K27me3, H3K9me3) and ATAC-seq data, together with published ChIP-seq (H3K27ac, H3K4me1, H3K4me3 and CTCF) (Jiang et al. 2017; Walsh et al. 2017) datasets, we categorized chromatin into six states for the adult mouse cortex (Fig. 2H left). We then calculated the enrichment of upregulated and downregulated genes, along with their corresponding anchors, in each of the chromatin state respectively. Our results revealed that upregulated genes and their corresponding anchors were primarily enriched in the enhancer regions (2, Enh; 3, EnhLo), characterized by H3K27ac, H3K4me1, and ATAC-seq signals (Fig. 2H right). Meanwhile, downregulated genes and their corresponding anchors were predominantly enriched in active gene promoters (1, Tss), featured with H3K4me3 and other open chromatin signals (Fig. 2H right). Consistent results were revealed (Fig. S5H) when we performed additional ChromHMM analysis using different sets of published ChIP-seq (ENCODE Project Consortium 2012; Hagelkruys et al. 2022; Mo et al. 2015) and ATAC-seq data (Stroud et al. 2020).

In summary, we discovered that the patterns of chromatin spatial interaction associated with upregulated genes markedly differed from those associated with downregulated genes. The upregulated genes, which exhibit more and stronger chromatin interactions, are primarily under the remote control of enhancers (Fig. 2I left, Fig. S5I left). Conversely, downregulated genes appear to be regulated by chromatin interactions among relative adjacent genes (Fig. 2I right, Fig. S5I right). These distinct regulatory patterns provide insight into the unique gene regulation signatures in adult cortex following prenatal exposure to BPA.

#### Alterations of DNA methylation in adult cortex after prenatal BPA exposure

**Fig. 3:**
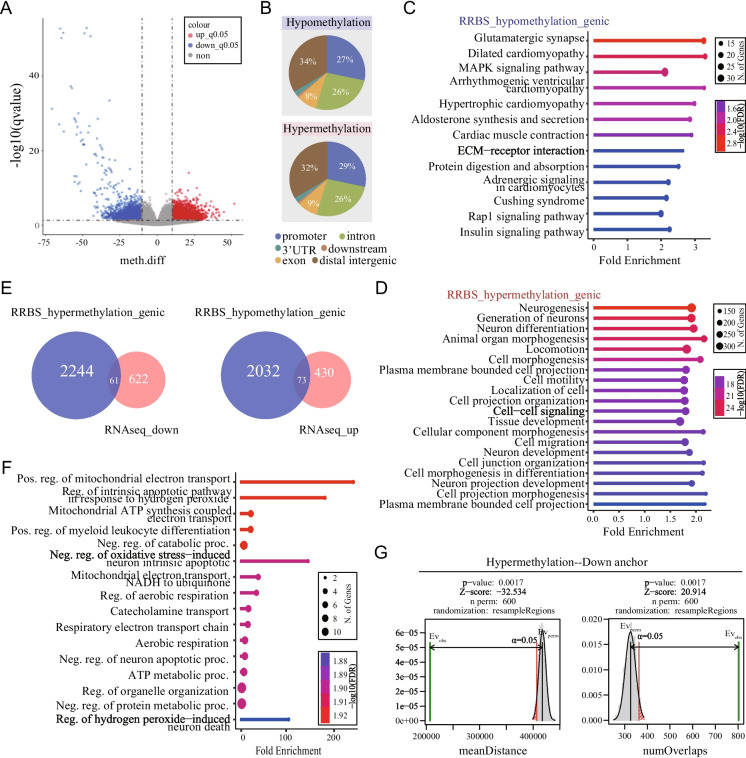
Alterations of DNA methylation in adult cortex after prenatal BPA exposure. **(A)** Volcano plot of DMSs in cortex of adult male offspring. Red, hypermethylated sites; blue, hypomethylated sites. BPA vs. Ctrl, q-value < 0.05 and methylation difference > 10%, n = 3/group. **(B)** Genome annotation pie-plot of the hypo- (Top) and hyper- (Bottom) methylated sites. ShinyGO analysis (“Biological Process”) of hypo- **(C)** and hyper- **(D)** methylated genes. **(E)** Overlap between down-regulated genes and hypermethylated genes (Left). Overlap between up-regulated genes and hypomethylated genes (Right). **(F)** ShinyGO analysis (“Biological Process”) of the overlapping hypermethylated and downregulated genes. **(G)** Permutation test shows mean distance (Left) and number of overlaps (Right) between DNA hypermethylated sites and anchors interacting with the promoters of downregulated genes

DNA methylation is one of the most extensively studied repressive epigenetic marks. It has been reported that BPA can influence DNA methylation thus affect the expression of neuronal genes (Alavian-Ghavanini et al. 2018; Kundakovic et al. 2015). To study whether DNA methylation contributed to gene dysregulation, we performed RRBS to profile genome-wide alterations of DNA methylation in adult cortex from both the BPA and Ctrl groups. We identified a total of 10,136 differentially methylated sites (DMSs), comprising 5,329 hypermethylated and 4,807 hypomethylated sites (q < 0.05, difference > 10%, n = 3/group) (Fig. 3A, Table S3). The alterations on the hypomethylation sites were more pronounced (Fig. 3A). Notably, genome annotation of DMSs revealed that 61% hypomethylated and 64% hypermethylated sites were gene-associated, located in the promoter, intron, and exon regions (Fig. 3B). GO analysis showed that hypomethylated genes were enriched in pathways of glutamatergic synapse and cell signaling (Fig. 3C), while hypermethylated genes were enriched in the pathways of neurogenesis, neuron differentiation and cell projection (Fig. 3D).

Despite the modest overlap between genes associated with DMSs and DEGs from RNA-seq (Fig. 3E), GO analysis of the 61 overlapping hypermethylated and downregulated genes showed significantly enrichment in pathways associated with mitochondrial functions, notably the energy respiratory chain (Fig. 3F). Meanwhile, 73 up-regulated genes were overlapped with hypomethylated genes, but no enriched pathway were identified in this group. Further analysis revealed that, compared to chromatin interaction anchors for all gene promoters, a significantly higher number of overlaps and a shorter mean distance were observed between DNA hypermethylation sites and regions (anchors) interacted with the promoters of downregulated genes (Fig. 3G). These findings suggest that prenatal BPA exposure leads to alterations in DNA methylation within the cerebral cortex of adult offspring, and DNA hypermethylation might partially account for the observed gene downregulation.

#### Alterations of repressive histone modifications in adult cortex after prenatal BPA exposure

**Fig. 4:**
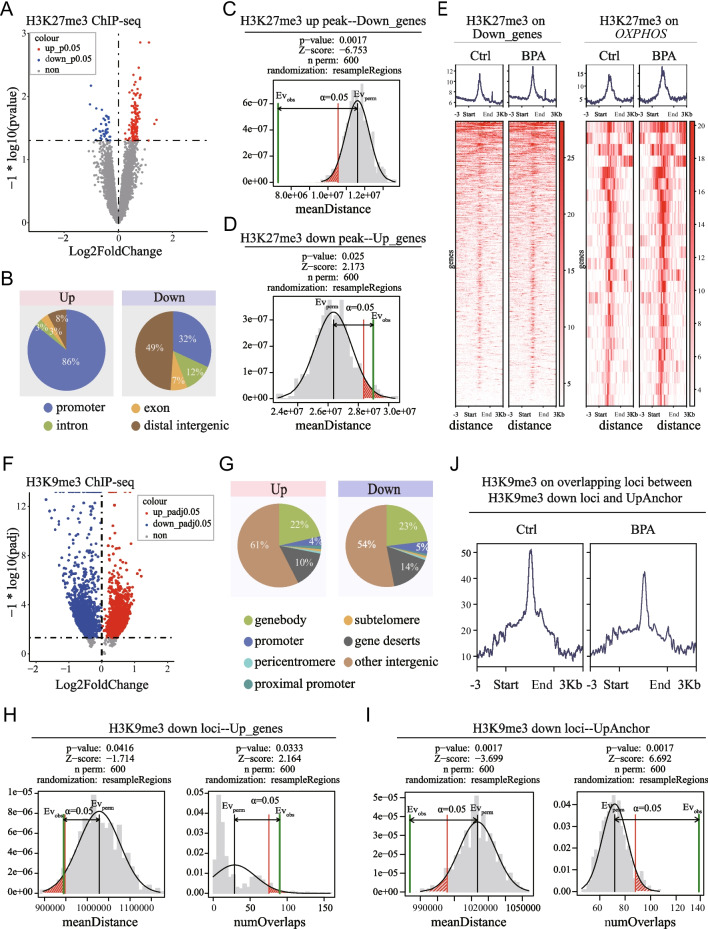
Alterations of repressive histone modifications in adult cortex after prenatal BPA exposure. **(A)** Volcano plot of differential H3K27me3 modification sites. Red, enhanced modification; blue, decreased modification. BPA vs. Ctrl, *P* < 0.05, n = 4/group. **(B)** Genome annotation pie-plot of the increased (Left) and decreased (Right) H3K27me3 modification sites. **(C)** Permutation test shows mean distance between increased H3K27me3 peaks and the TSS of downregulated genes. **(D)** Permutation test shows mean distance between decreased H3K27me3 peaks and the TSS of upregulated genes. **(E)** Heatmap of H3K27me3 signals on ± 3 Kb of Down_genes (Left) and OXPHOS gene promoters (Right). In each group, the signal of three biological replicates were merged. The gradient color bar indicates the signal strength. **(F)** Neurons of adult male cortex were enriched by FANS sorting for H3K9me3 ChIP-seq. Volcano plot of differential H3K9me3 modification sites were shown. Red, enhanced modification; blue, decreased modification. BPA vs. Ctrl, for differential H3K9me3 sites, cut-off was set as: *P*adj < 0.05, basemean > 50, n = 4 Ctrl/5 BPA. **(G)** Genome annotation pie-plot of the increased (Left) and decreased (Right) H3K9me3 sites. **(H)** Permutation test shows mean distance (Left) and number of overlaps (Right) between decreased H3K9me3 loci and TSS of upregulated genes. **(I)** Permutation test shows mean distance (Left) and number of overlaps (Right) between decreased H3K9me3 loci and anchors interacting with the promoters of upregulated genes. **(J)** H3K9me3 signals at the overlapping regions between H3K9me3 decreased loci and anchors interacting with the promoters of upregulated genes

In addition to DNA methylation, repressive histone modifications such as H3K27me3 and H3K9me3 also mediate long-term effects of BPA (Fatma Karaman et al. 2019; Senyildiz et al. 2017). We, therefore, performed H3K27me3 ChIP-seq on adult cortex from both BPA and Ctrl groups. On average, we detected 10,581 H3K27me3 peaks per sample. however, only a small percentage (1.6%) of these peaks were altered in the BPA compared to the Ctrl group. Among them, 125 peaks showed increased H3K27me3 occupancy in the BPA group (*P* < 0.05, n = 4/group) (Fig. 4A, Table S4), with 86% of these peaks located on gene promoters (Fig. 4B, left). Only 41 peaks were downregulated in the BPA group (*P* < 0.05, n = 4/group), and 32% on the gene promoters (Fig. 4B, right). Subsequently, we investigated the association between differential H3K27me3 peaks and DEGs. Permutation tests revealed that the location of upregulated H3K27me3 peaks were significantly closer to the TSS of downregulated genes (Fig. 4C). Conversely, downregulated H3K27me3 peaks were significantly further away from the TSS of upregulated genes (Fig. 4D). Moreover, H3K27me3 signals peaked at the TSS of downregulated genes, and these signals were more pronounced in the BPA compared to the Ctrl group (Fig. 4E, left), especially for the OXPHOS genes (Fig. 4E, right). These findings suggest that H3K27me3 might regulate gene expression by directly repressing the promoter activity of downregulated genes in adult cortex after BPA exposure.

To improve detection sensitivity and minimize interference from various cell types in the brain, we applied fluorescence activated cell nuclei sorting (FANS) to isolate NeuN + nuclei from the adult cortex for H3K9me3 ChIP-seq. Differential analysis, performed using diffReps with a window size of 1 Kb, identified significant alterations in H3K9me3 occupancy in response to prenatal BPA exposure (*Padj* < 0.05, n = 4 Ctrl/5 BPA) (Fig. 4F, Table S5). Unlike H3K27me3, genome annotation showed that the majority of differential H3K9me3 sites were located in gene deserts and other intergenic regions. Only about 25% of these sites were gene-related for both upregulated and downregulated sites (Fig. 4G), implying H3K9me3 might primarily function on distal regulatory elements. Indeed, when we examined the association between downregulated H3K9me3 loci and upregulated genes, we only detected mild enrichment in terms of distance or overlapping number compared to the background (Fig. 4H). However, permutation tests revealed a significantly higher number and a shorter mean distance between downregulated H3K9me3 loci and regions (anchors) interacting with upregulated genes (Fig. 4I). Moreover, H3K9me3 signals were notably lower in the BPA group compared to the Ctrl group at these overlapping regions (Fig. 4J). These findings suggest that the loss of H3K9me3 occupancy on distal regulatory regions may contribute to the upregulation of gene expression in adult cortex after prenatal BPA exposure.

## Discussion

**Fig. 5:**
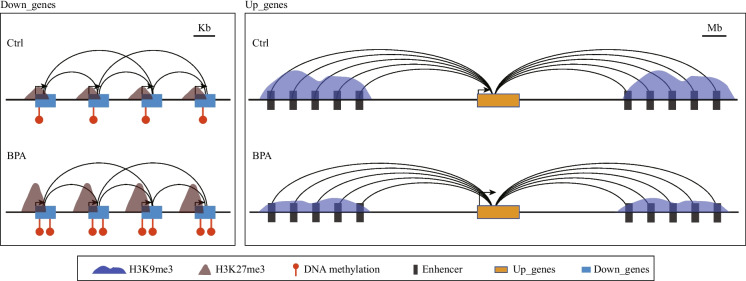
Schematic diagram displays differential epigenetic regulations for upregulated and downregulated genes. The upregulated genes, which exhibit more and stronger chromatin interactions, are primarily regulated by the decreased H3K9me3 modification on the distal enhancers (Right). The downregulated genes were regulated directly by the increased DNA methylation and H3K27me3 modification at the promoter regions (Left)

The US Environmental Protection Agency (USEPA) has set a reference dosage (RfD) for BPA of 50 µg/kg bw/day (Shelnutt et al. 2013), which is estimated as a safe daily oral exposure level for humans. In this research, we chose a more conservative dose of 40 µg/kg bw/day and employed a mouse model for prenatal BPA exposure to investigate the molecular mechanisms of neurological dysregulation in the brains of adult offspring. Our findings revealed that prenatally BPA exposure resulted in significant transcriptional dysregulation in the adult brain, although with subtle behavioral changes. This effect was notably more pronounced in male offspring. Transcriptomic analysis indicated that the upregulated genes predominantly influenced neuronal functions. In contrast, the downregulated genes were primarily associated with energy pathways, including an array of OXPHOS genes. Further evidence suggested compromised mitochondrial function. Additionally, we identified distinct chromatin interaction patterns correlating with upregulated and downregulated genes (Fig. 5). The upregulated genes were characterized by more robust chromatin interactions from various remote enhancers, whereas the downregulated genes formed chromatin interactions among neighboring genes. Moreover, we discovered that increased DNA methylation and H3K27me3 were involved at the promoter regions of the downregulated genes. Meanwhile, a decrease in H3K9me3 occupancy was noted on the distal regulatory regions of the upregulated genes. To summarize, our research presents compelling evidence of sex-specific gene dysregulation in the adult offspring's brain following prenatal BPA exposure. It also highlights unique higher-order chromatin regulatory patterns and underscores the intricate interplay among multiple epigenetic modifications driving this process.

### Sex difference in transcriptional alteration of adult offspring’s brain following prenatal BPA exposure

Sexual dimorphism in response to prenatal stress has been observed in numerous studies (Barrett and Lessing 2021). Clinical evidence suggests that male fetuses tend to exhibit higher susceptibility to adverse pregnancy outcomes compared to females, often described as “male fragility” (Barrett and Lessing 2021). This trend extends to animal studies. Our study, along with others has demonstrated that male offspring are more susceptible (Thongkorn et al. 2021), although controversial report also exists regarding females (Arambula et al. 2018). The mechanisms underlying these sex differences were largely unknown. BPA, as an endocrine disruptor, is known to interfere with the function of hormone receptors including estrogen receptor (ER) and others (Kundakovic et al. 2013). The differential expression of hormone receptors in response to BPA may contribute to sex differences. For instance, at exposure to a low dose of BPA, the expression of estrogen-related receptor gamma (ESRRG) decreased in male placenta, while increased in females (Zou et al. 2022). In addition, a key finding suggests that O-GlcNAc transferase (OGT) in the placenta plays a crucial role in determining resilience to prenatal stress. OGT levels were found to be lower in male placentas compared to females, and further decreased after prenatal stress (Howerton and Bale 2014). This could potentially account for the increased vulnerability of males subjected to prenatal adversities.

### Impaired energy metabolism in adult brain after prenatal BPA exposure

The human brain accounts for 20% of the body's total oxygen consumption. Neurons predominantly rely on aerobic metabolism and procure energy substrates from astrocytes. These substrates are then transported to the mitochondria where they undergo oxidative phosphorylation and produce ATP (Jha and Morrison 2020). Our study implied a potential impairment in energy metabolism in the adult brain following prenatal BPA exposure, particularly pronounced in male offspring. This aligns with findings from a rat model in which reduced glucose metabolism is reported in prefrontal cortex and hippocampus of offspring in adulthood following prenatal BPA exposure (Xu et al. 2019). Moreover, region-specific alterations in oxidative energy metabolism were reported in fetal brain of sheep (Guignard et al. 2022). These findings suggest that prenatal BPA exposure may trigger enduring changes in mitochondrial function, potentially compromising neuronal functions in adulthood.

### Unique patterns of epigenetic regulation of differential expressed genes

In our study, we explored the interplay of DNA methylation, histone modifications, and higher-order chromatin interactions in the regulation of gene transcription following prenatal BPA exposure. Our model revealed multiple long-range interactions connecting enhancers and upregulated genes, which mirrors the sophisticated regulatory patterns known to govern neuronal genes (Beagan et al. 2020). Moreover, de-repression as indicated by decreased H3K9me3 was noticed at remote enhancers, which could contribute to the observed upregulation of genes after BPA exposure. These findings echo the work of Amita Bansal and colleagues, who reported remote chromatin interactions between DMSs and DEGs in human amniocytes following prenatal BPA exposure (Bansal et al. 2019). In addition to enhancer-promoter interactions, promoter-promoter interactions represent another type of spatial regulome that coordinates transcriptional control among genes (Schoenfelder 2019). Our data showed a substantial proportion (80%) of chromatin interactions associated with the promoters of downregulated genes. Although these downregulated genes and their corresponding anchors were significantly enriched in open chromatin under normal conditions, increased DNA methylation and H3K27me3 were observed following BPA exposure, which could potentially explain the diminished expression of this subset of genes.

### Limitations and future perspectives

Several questions remain to be addressed in this study. Firstly, there is a lack of direct link between observed molecular alterations and any notable behavioral changes, suggesting potential compensatory mechanisms in the brain to maintain homeostasis despite molecular disturbances. However, this may increase susceptibility to future adverse events, as noted in previous research on early-life stress. Therefore, the observed changes in energy metabolism-related genes in our study could potentially have implications manifesting later in life or upon subsequent challenges, making this an interesting topic for future study. Secondly, while we detected distinct patterns of epigenetic modulation after prenatal BPA exposure, it is not yet clear whether these modifications are directly responsible for the observed changes in gene transcription. To confirm the cause-and-effect relationship, it would be necessary to investigate whether manipulation of these epigenetic changes could normalize the altered gene expression profiles. This could potentially be achieved by using small molecules that interfere with the activity of enzymes for these epigenetic modifications. However, achieving specificity for certain brain region or genes would be challenging. Moreover, as the role of epigenetics in mediating long-lasting effects of early-life adversity is well-accepted (Goyal et al. 2019), it would be interesting to check whether the observed epigenetic changes in the adult brain are already present in early developmental stages. Lastly, although our RNA-seq analysis revealed the enrichment of downregulated genes in the energy metabolic pathway, and a reduction of ATP was detected in the brains of the BPA group, more direct evidence would be necessary to substantiate these findings. Future investigations are crucial to elucidate mitochondrial function and metabolic changes following prenatal BPA exposure.

In conclusion, while our study provides valuable insights into the potential epigenetic mechanisms underlying the long-term effects of prenatal BPA exposure in the adult brain, further research is needed to fully elucidate these complex processes and their implications for neurological health.

## Materials and methods

### Animals

C57BL/6Slac mice were purchased from Shanghai SLAC Laboratory Animal Co. Ltd, Shanghai, China, and maintained on a 12–12 h light/dark cycle with ad libitum access to water and food. All animal work was approved by the Animal Care and Use Committee of Shanghai Medical College of Fudan University.

### Procedure of BPA exposure

Female mice aged 3–6 months were utilized for timed-pregnancy breeding, following a mating protocol of two females to one male per cage. Breeding cages were set up between 5:00 p.m. and 6:00 p.m., and the presence of vaginal plugs was checked the following morning (GD 0.5). Plugged females were then randomly assigned to two groups and administered either corn oil (Ctrl) or 40 μg/kg bw/day of BPA by oral gavage from GD 0.5 to GD 13.5. To obtain adult offspring, the pregnant mice were separated into individual cages 1–3 days prior to parturition and offspring were weaned at postnatal day 21 and group-housed in cages of 3–5 mice. In total, 12–14 litters per group were collected in this study.

### RNA-seq library preparation

RNeasy Lipid Tissue Mini Kit (Qiagen Cat. No. 74804) was used to extract total RNA. In brief, adult prefrontal cortex was collected and homogenized in QIAzol Lysis Reagent. Then the sample was mixed thoroughly with chloroform and centrifuged at 12000 g at 4 ℃ for 15 min. The upper aqueous phase was mixed with 70% ethanol and then transferred to RNeasy Mini spin column and eluted in RNase-free water. The concentration of RNA was measured by Nanodrop. A total of 1 μg RNA was used for mRNA library preparation and sequencing with MGISEQ-2000 set single-end, 50 bp (SE50) (BGI Genomics, China).

### ChIP-seq library preparation

Cortex homogenate was used for H3K27me3 ChIP. As for H3K9me3, Nuclei were extracted from the cortex of male offspring and neuronal (NeuN +) nuclei were enriched by FANS sorting using MoFlo Astrios EQ cell sorter (Beckman Coulter). Native ChIP was performed as described (Jiang et al. 2017). Briefly, the chromatin was digested with MNase at 28 ℃ for 10 min to obtain mononucleosomes and incubated with anti-H3K9me3 (Abcam AB8898) or anti-H3K27me3 (Millipore, 07–449) antibody at 4 ℃ overnight. The immunoprecipitated complexes were captured by protein A/G magnetic beads (Thermo Scientific, 88803) and washed with low-salt buffer, high-salt buffer, Lithium Chloride buffer and TE buffer. ChIP DNA was then eluted in elution buffer and incubated with RNase A, followed by proteinase K incubation. Finally, ChIP DNA was purified using SPRI magnetic beads (Beckman, B23318).

For ChIP DNA library preparation, End-repairing (Lucigen Corporation, ER0720) and A-tailing (Lucigen Corporation, KL11101K) was performed and then ChIP DNA was ligated (Lucigen Corporation, LK0750H) with Y-adaptor (Vazyme, N802) and subjected to PCR amplification (Vazyme, N618-01). Library DNA was size-selected with SPRI beads and sent to GENEWIZ,China, for deep sequencing with Novaseq set paired-end, 150 bp (PE150).

### RRBS library preparation

Nuclei were extracted from cortex homogenates and incubated with RNaseA (Sigma R6513) at 37 ℃ for 15 min, followed by proteinase K (Sigma P2308) incubation at 52 ℃ overnight. The samples were mixed with phenol/chloroform (1:1) (Solarbio P1021), and centrifugated. The supernatant was collected and then precipitated by isopropanol. The product was washed in 70% ethanol, air dried, and dissolved by buffer EB (Qiagen 19086). A total of 1 μg DNA was used for RRBS library preparation and sequenced with Novaseq set paired-end, 150 bp (PE150) (Novogene, China). Briefly, unmethylated lambda DNA was added into the genomic DNA (gDNA) and incubated with MspI enzyme to obtain 200 to 1000 bp fragments. The DNA fragments were then converted by bisulfite using the EZ DNA Methylation-Gold Kit (Zymo Research, United States).

### TEM sample preparation

Prefrontal cortex of male offspring was freshly isolated, and immediately transferred on a hardboard with pre-cooled 2.5% glutaraldehyde. Tissue sample was carefully trimmed into 0.5–1 mm^3^ and mixed with 1 mL of pre-cooled 2.5% glutaraldehyde. The mixture was then placed in 4 ℃ for at least 2 h and submitted to the Electron Microscopy Core Laboratory in the School of Basic Medical Sciences, Fudan University.

### Measurement of ATP

Half cortex of male offspring was isolated and homogenized with Lysis buffer (0.32 M Sucrose, 5 mM CaCl_2_, 3 mM Mg(Ace)_2_, 0.1 mM EDTA, 10 mM Tris–HCl, 0.1% NP40), followed by centrifugation at 12000 g at 4 ℃ for 5 min. The supernatant was used for ATP measurement using ATP Detection Assay Kit (Beyotime, S0026). TECAN Infinite M200 illuminometer was used to record the luminescence.

### Real-time RT-PCR

RNA was converted into cDNA using iScript™ cDNA Synthesis Kit (Bio-Rad, 1708891). PCR reaction mix were assembled using Power SYBR™ Green PCR Master Mix (Thermo, 4368702) and amplified on Thermo Fisher Scientific Applied Biosystems QuantStudio5. *Gapdh* was used as the reference gene. Primers used were provided in Table S6.

### Data analysis

#### RNA-seq

Quality control of the raw data were conducted by FastQC and Trim-galore with adaptor and low-quality reads filtered out. The clean data were then aligned to reference genome mm10 (UCSC) through HISAT2. Then SAMtools v1.6 were used to convert SAM files to BAM files, sort and build the alignment file index. The FeatureCounts v1.6.3 was used for gene count with parameters set as: -t exon -g gene_id and DESeq2 was used for differential analysis with P < 0.05 as cutoff for significant genes. Gene Otology analysis was performed through ShinyGO 0.77 (GO Biological Process).

#### Integrative analysis of bulk RNA-seq and scRNA-seq

The published scRNA-seq (GSE124952) (Bhattacherjee et al. 2019) data of adult mouse prefrontal cortex was incorporated in the cell-type enrichment analysis of DEGs from our adult male RNA-seq datasets, with only the data of saline group used. DEGs contains two gene sets, “Up_genes” and “Down_genes”, that were analyzed separately. Seurat’s Dimplot function was used to generate a t_SNE plot. The average expression of genes in “Up_genes”or “Down_genes” gene set (only detected in scRNA-seq data) in each cells was calculated and scaled by DoHeatmap function. The feature expression was visualized in the t_SNE plot by R packages ggplot2. Then marker genes of each cell cluster were identified by FindAllMarkers function with parameters setting as: only.pos = TRUE, min.pct = 0.25, logfc.threshold = 0.5. Enrichment score of the two gene sets in marker genes list of each cell cluster were calculated using R packgage GeneOverlap (Peña et al. 2019).

#### Hi-C analysis

Hi-C datasets were downloaded from GSE99363 (Jiang et al. 2017). Quality control of the raw data were conducted by FastQC and Trim-galore with adaptor and low-quality reads filtered out. Clean data were mapped to mouse genome mm10 (UCSC) by HICUP (v0.8.0) and HiC-Pro (v2.11.4) was used to generate valid pairs and build normalized contact matrices in 20-Kb-resolution. Fit-Hi-C (v2.0.8) was used to screen out the significant contacts with q-value < 0.01. Gene-associated significant contacts were defined as either anchor of the contacts overlapping with genes and identified by the “intersect” command of Bedtools (version 2.30.0) in default settings. For each gene, we calculated the cumulative interaction score of significant contacts, referring to its intensity of contacts. By ranking cumulative interaction scores for genes, we were able to identify the 5% top genes with the highest cumulative interaction scores. To test whether up- and down- regulated genes were significantly enriched in these 5% top genes with highest cumulative interaction score, we first randomly selected 501 genes (when compared with Up_genes) or 681 genes (when compared with Down_genes) from the background genes and calculated the overlapped genes between the selected genes and the top 5% genes. This process was repeated 10000 times to obtain the frequency distribution of permuted number of overlapped genes and the P-value of overlapped genes greater than 87 or less than 4 were calculated. ChromHMM v1.23 was used for unsupervised segmentation of the mouse genome into 6 states based on our own ATAC-seq, H3K27me3, and H3K9me3 ChIP-seq, together with online published H3K27ac (GSE99363), CTCF (GSE99363) (Jiang et al. 2017), H3K4me1 (GSE90020) and H3K4me3 (GSE90020) (Walsh et al. 2017) ChIP-seq datasets. These states were annotated according to the published article (van der Velde et al. 2021). Each contact contained two anchor bins. Once a gene was successfully mapped to an anchor of the contacts, the corresponding other anchor was termed gene associated anchor. The coordinates of genes or their corresponding anchors were used to assess the enrichment of these candidate regions in the 6 chromatin states. Notably, the color key referred to the column specific color scale, which subtracted the minimum value in the column and were then divided by the maximum column value.

#### RRBS

Data alignment was performed by Bowtie2 with default settings. Duplicated reads were then filtered out by “deduplicate_bismark” function of Bismark (version 0.22.1). The methylation information were extracted by “bismark_methylation_extractor” (–no_overlap –ignore_r2 2 –comprehensive) and used for calculation of the bisulfite conversion rate based on methylation level of spiked-in lambda DNA. The differential analysis was carried out MethylKit R package. Briefly, the list with methylation information was read by “methRead” function with mincov set to 3 and then filtered by “filterByCoverage” with parameters setting as: lo.count = 5,lo.perc = NULL, hi.count = NULL, hi.perc = 99.9. The differential methylation was calculated by the function “calculateDiffMeth” and defined as: qvalue > 0.05 and meth.diff >|10|.

#### ChIP-seq

Data were mapped to genome mm10 (UCSC) by Bowtie2 v2.4.1 with default settings. H3K27me3 peaks were called by Macs2 with parameters set as: -f BAM –broad -g mm. Differential analysis of H3K27me3 peaks was performed using “DiffBind” (version 3.4.11). The consensus peaks were identified by dba.count and dba.peakset functions, and difference were measured by dba.analyze with “DBA_DESEQ2” method. Differential analysis of H3K9me3 signals was conducted by “DiffReps” (version 1.55.6) (Shen et al. 2013) with the parameters setting as: -pval 0.001 -frag 150 -window 1000 -me gt. Deeptools was used to create the protmaps of ChIP-seq signals (Ramírez et al. 2014) with parameters of “computeMatrix scale-regions” setting as: –regionBodyLength 5000 -a 3000 -b 3000 –skipZeros. To evaluate statistical significance of the distance between genes and ChIP-seq peaks, permutation tests were performed by “regioneR” (version 1.26.1) (Gel et al. 2016) with 600 times sampling. The background was set as all genes, while the evaluate function set as “meanDistance”.

## Supplementary information

Below is the link to the electronic supplementary material.

Supplementary file1 (DOCX 1.91 MB)

Supplementary file2 (DOCX 18.0 KB)

Supplementary file3 (XLSX 1417 KB)

## Data Availability

All raw and processed sequencing data have been submitted to the Gene Expression Omnibus (GEO) under the accession number GSE249016.

## Abbreviations

*BPA*: Bisphenol A
*DEGs*: Differentially expressed genes
*RRBS*: Reduced Representation Bisulfite Sequencing
*OXPHOS*: Oxidative phosphorylation
*GO*: Gene Ontology
*DMSs*: Differentially methylated sites
*DMRs*: DNA methylated regions
*FANS*: Fluorescence activated cell nuclei sorting
