# Supplementary material for: Distinct epigenetic modulation of differentially expressed genes in the adult mouse brain following prenatal exposure to low-dose bisphenol A
Source: Cell Biol Toxicol. 2024 May 22;40(1):37. doi: 10.1007/s10565-024-09875-4 (PMC11111541; doi:10.1007/s10565-024-09875-4)
Supplement: Supplementary file 1 — Supplementary file1 (DOCX 1.91 MB) [file 10565_2024_9875_MOESM1_ESM.docx]

**Supplemental Figures**

**Title:** Distinct epigenetic modulation of differentially expressed genes in the adult mouse brain following prenatal exposure to low-dose bisphenol A

**Journal:** Cell Biology and Toxicology

**Authors:** Jie Weng^1†^, Yue-yan Zhu^1†^, Li-yong Liao^1^, Xin-tong Yang^2^, Yu-hao Dong^1^, Wei-da Meng^3^, Dai-jing Sun^1^, Yun Liu^3^, Wen-zhu Peng^1*^, Yan Jiang^1*^

*^1^Institutes of Brain Science, State Key Laboratory of Medical Neurobiology and MOE Frontiers Center for Brain Science, Fudan University, 200032, Shanghai, China.*

*^2^Shanghai Medical college, Fudan University, 200032, Shanghai, China.*

*^3^The MOE Key Laboratory of Metabolism and Molecular Medicine, Department of Biochemistry and Molecular Biology, School of Basic Medical Sciences, Fudan University, 200032, Shanghai, China.*

^†^JW and YZ contributed equally to this work

^*^WP and YJ are co-senior authors

**Correspondence**: Yan Jiang, [Yan_jiang@fudan.edu.cn](mailto:Yan_jiang@fudan.edu.cn)


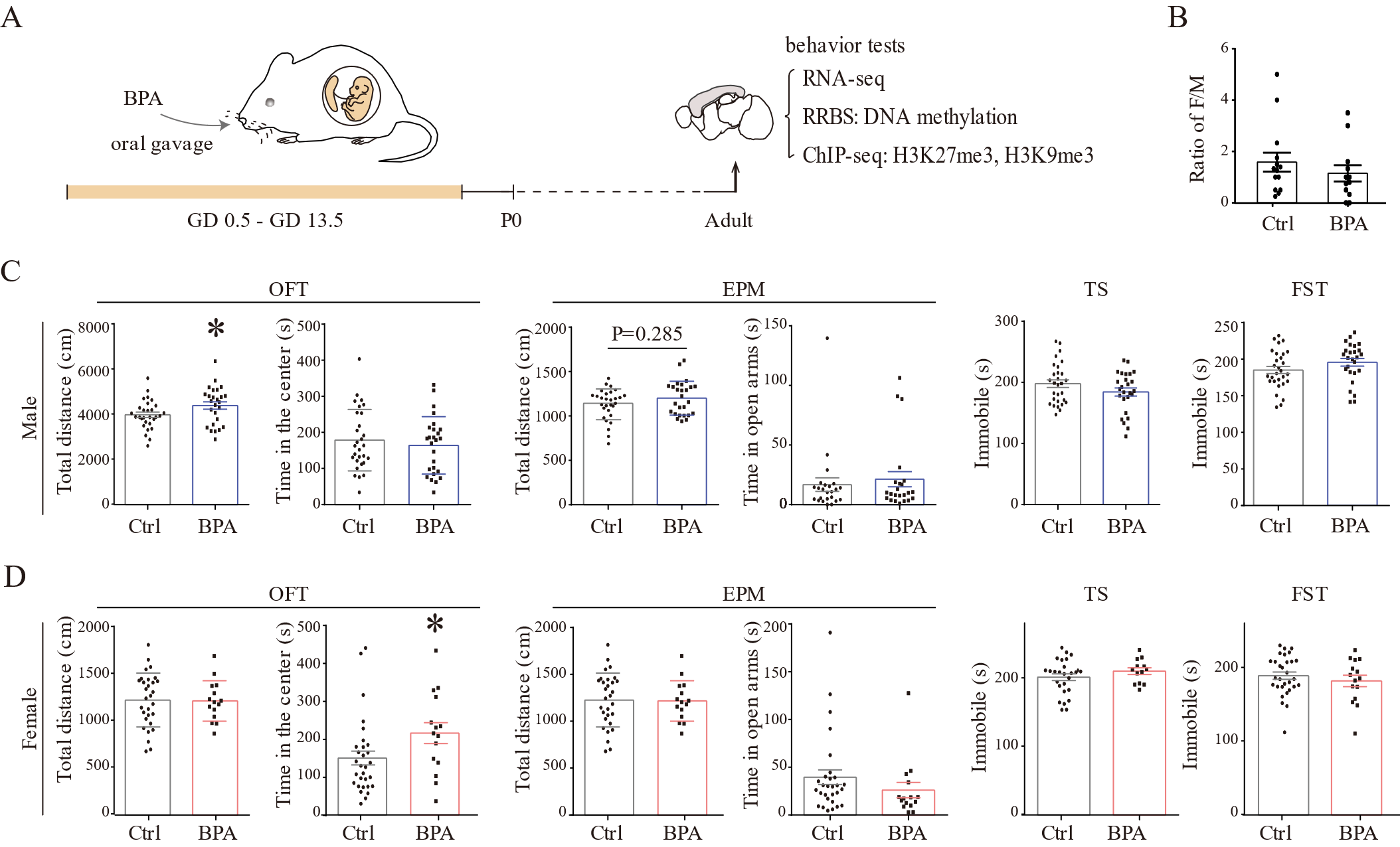


**Supplementary Figure 1. Behavioral performance of adult offspring following prenatal BPA exposure. (A)** Schematic timeline of experimental procedure. Pregnant mice were administrated with corn oil (Ctrl) or 40 ug/kg bw/day BPA by oral gavage from GD0.5 to GD13.5. Adult offspring were subjected to behavioral tests, and the brains were collected for transcriptomic and epigenomic profiling. **(B)** Sex ratio (female to male) of offspring. N = 12-14 litters/group. Mean ± SEM. Unpaired *t*-test, two-tailed. (**C**) Behavioral performance of male offspring. Total distance in open field was increased in the BPA group. No significant difference was detected in the elevated plus maze test, tail suspension test and forced swimming test. N = 26-29/group. Mean ± SEM. **P* < 0.05, Unpaired *t*-test, two-tailed. (**D**) Behavioral performance of female offspring. Female mice spent more time in the center. No significant difference was detected in the elevated plus maze test, tail suspension test and forced swimming test. N = 15-30/group. Mean ± SEM. **P* < 0.05, Unpaired *t*-test, two-tailed.


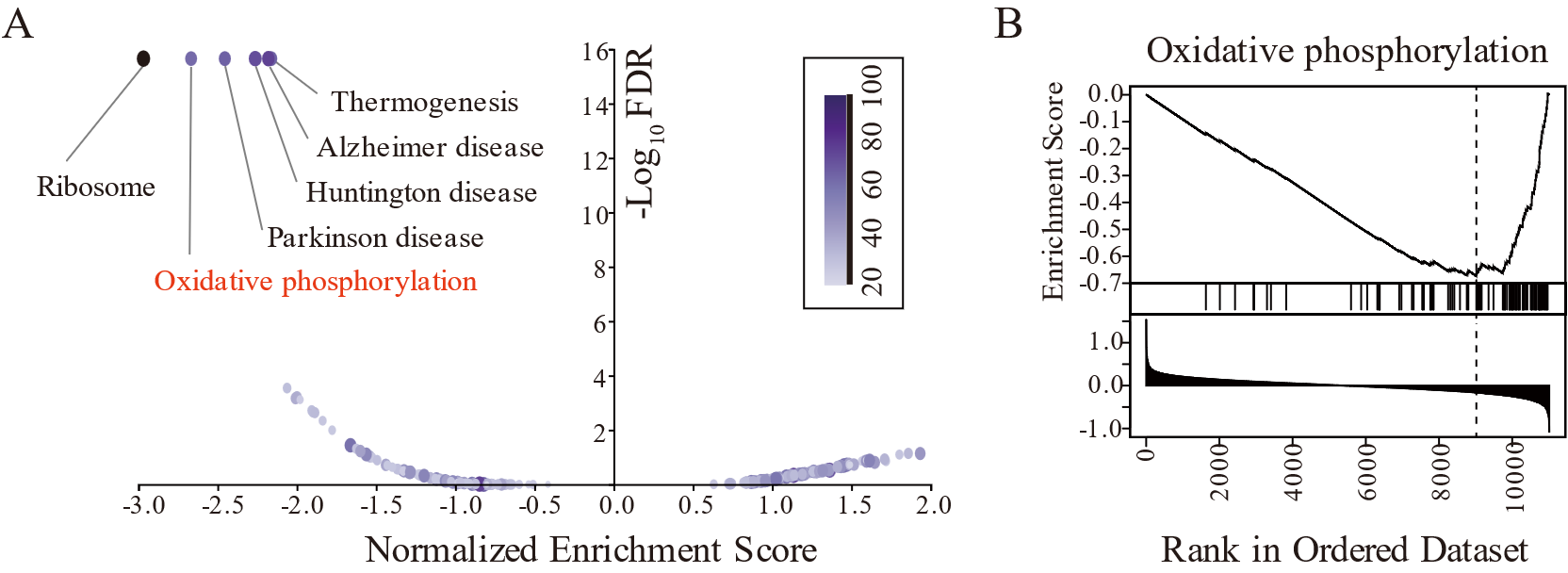


**Supplementary Figure 2. Decreased OXPHOS pathway in adult cortex after prenatal low-dose BPA exposure**. (**A**) Dot plot of Gene Set Enrichment Analysis (GSEA) KEGG pathway enrichment (BPA vs. Ctrl) shows the significantly enriched pathways. Color key for number of genes in each pathway. (**B**) Enrichment score (ES) plots for oxidative phosphorylation (Right). The KEGG functional database in WEB-based GEne SeT AnaLysis Toolkit (WebGestalt, https://www.webgestalt.org/) was used for GSEA.

**
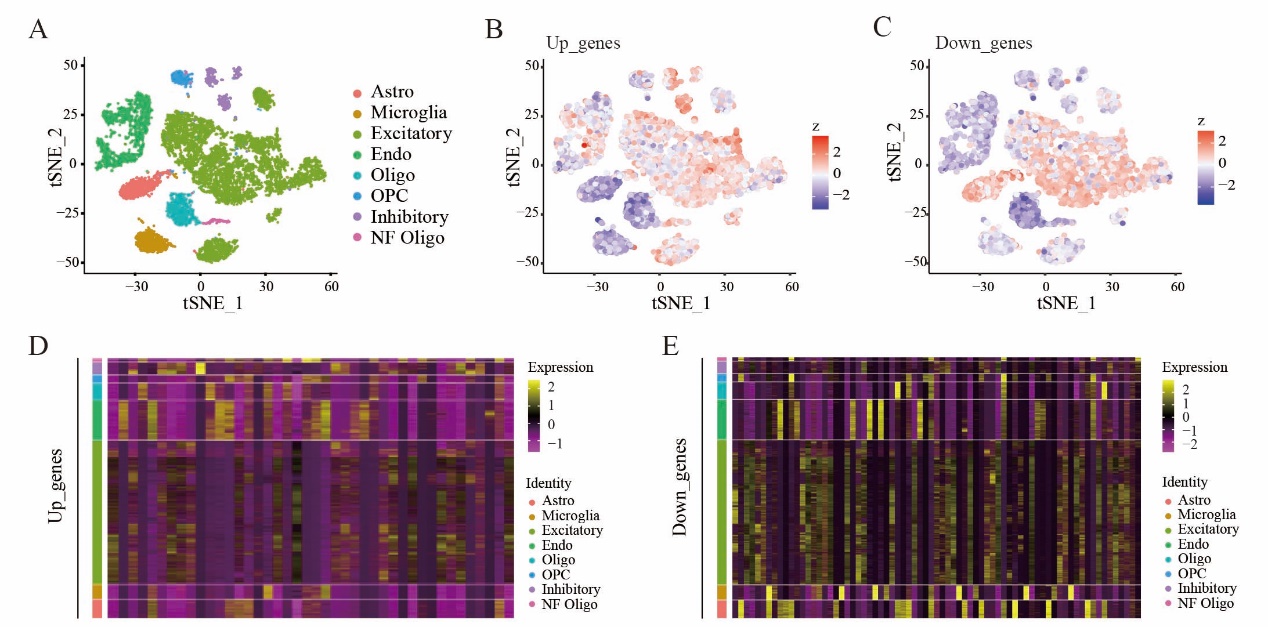
**

**Supplementary Figure 3. Enrichment of differentially expressed genes in different cell types.** **(A)** Scatterplots visualize the 8 clusters of cell types in the t-SNE map of published scRNA-seq data. The 8 cell clusters were: Excitatory neuron (Excitatory), Astrocyte (Astro), Oligodendrocyte (Oligo), Inhibitory neuron (Inhibitory), Microglia, Oligodendrocyte precursor cell (OPC), Endothelial cell (Endo). Newly formed Oligodendrocyte (NF Oligo). Scatterplots visualize the expression and distribution of “Up_genes” **(B)** and “Down_genes” **(C)** in the t-SNE map. Seurat’s DoHeatmap shows the individual gene expression level of “Up_genes” **(D)** and “Down_genes” **(E)** in each cell of the published scRNA-seq. Only genes detected in scRNA-seq were shown.


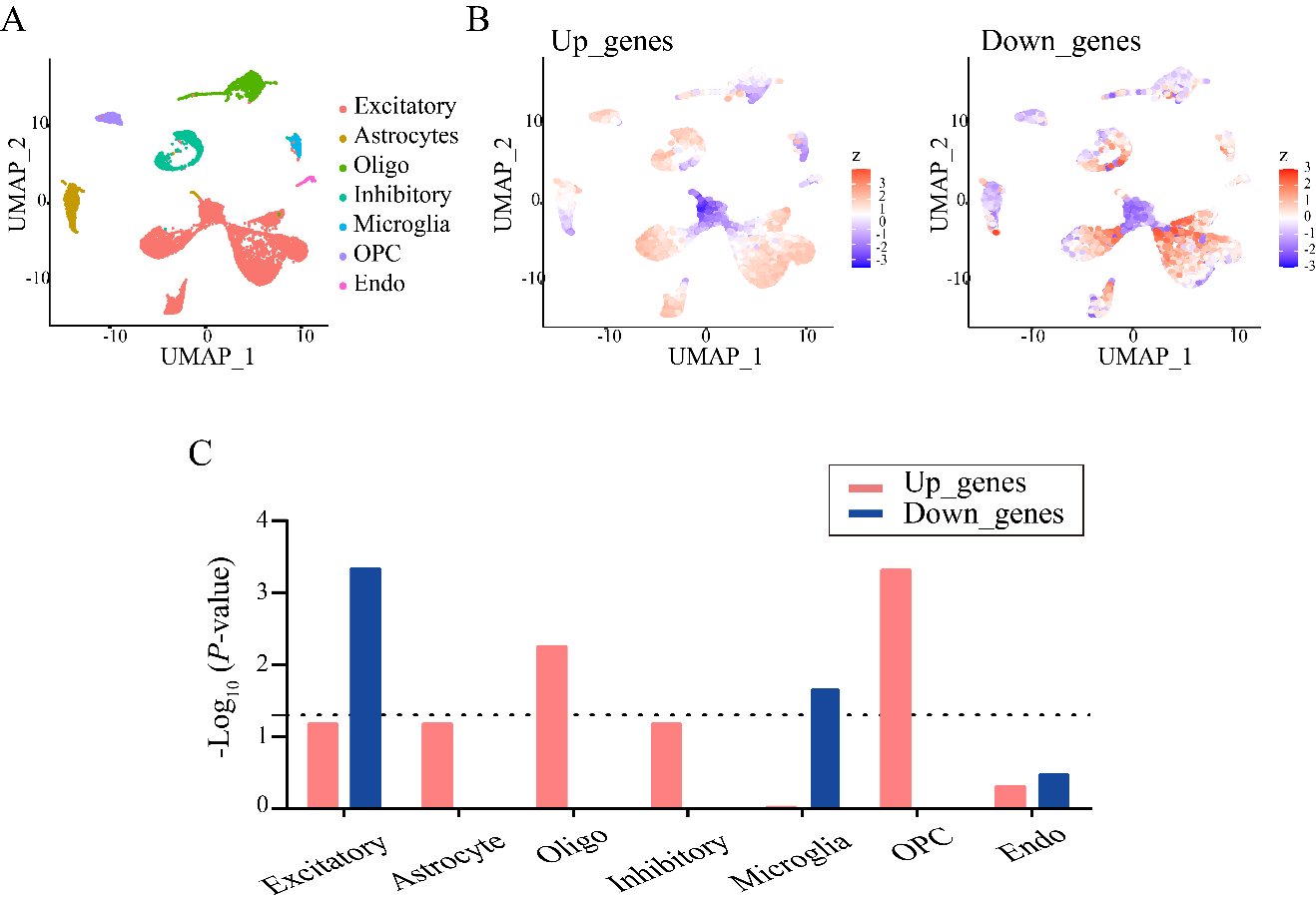


**Supplementary Figure 4.**:Additional analysis of cell type enrichment of DEGs using a different set of scRNA-seq data. (**A**) Scatterplots visualize the 8 clusters of cell types in the published scRNA-seq data (GSE211099). The 8 cell clusters were: Excitatory neuron (Excitatory), Astrocyte, Oligodendrocyte (Oligo), Inhibitory neuron (Inhibitory), Microglia, Oligodendrocyte precursor cell (OPC), Endothelial cell (Endo). (**B**) Scatterplots visualize the average expression of “Up_genes” (Left) and “Down_genes” (Right) in the scRNA-seq data. Both upregulated and downregulated genes had relatively high expression level in neuronal cells. (**C**) Enrichment analysis revealed that downregulated genes were primarily enriched in excitatory neurons and microglia, while upregulated genes were mainly enriched in oligodendrocytes and their precursors.


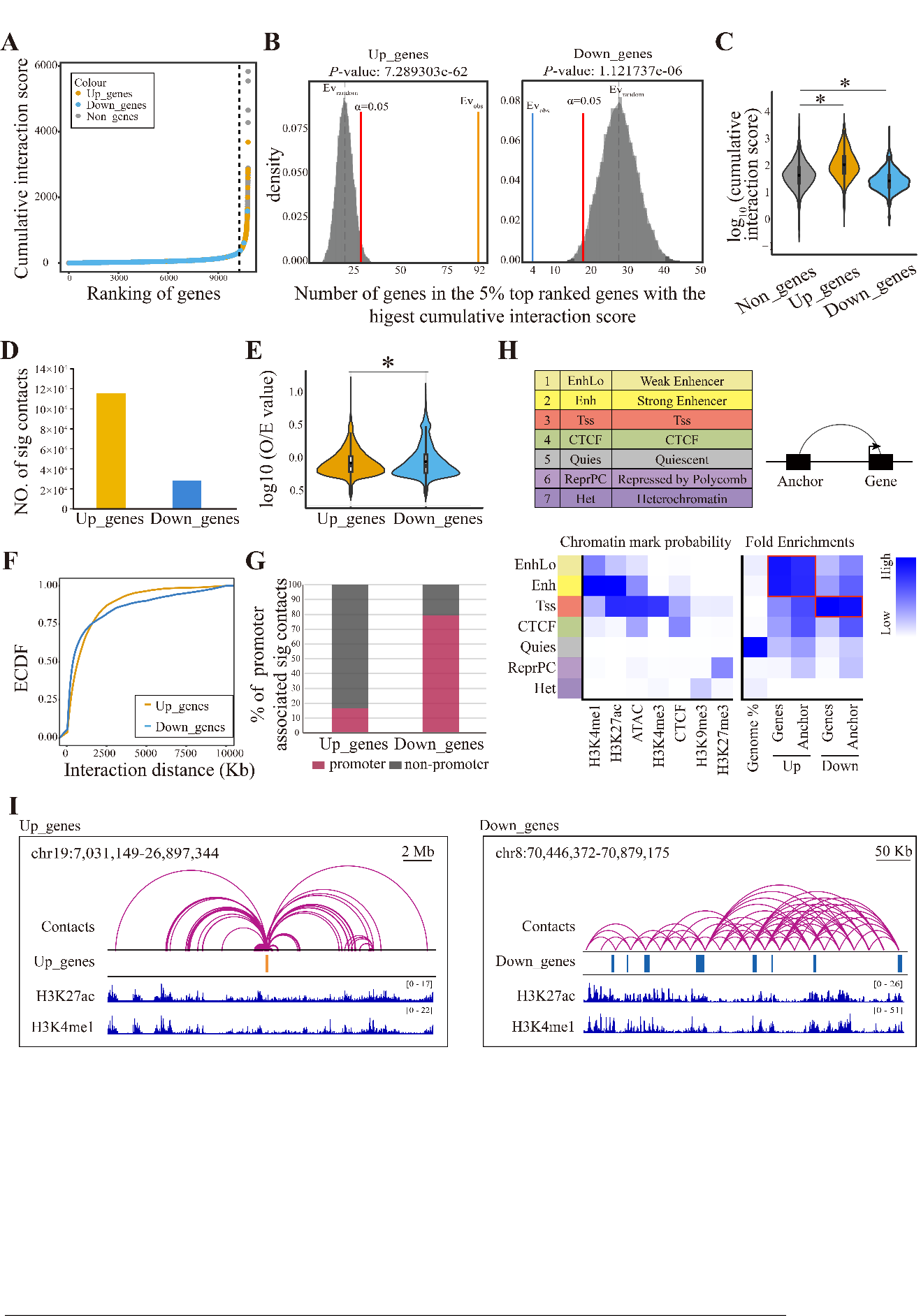


**Supplementary Figure 5. Additional analysis of 3D epigenomic signature of DEGs using different datasets.** **(A-G)** Validation of distinct chromatin interaction pattern of DEGs (Fig 2) using a different set of Hi-C data (GSE168524) **(A-C)** Upregulated genes were significantly enriched in the top 5% genes with highest cumulative interaction score, while downregulated genes significantly underrepresented among those top 5% genes. **(A)** the Gene-associated contacts were extracted out and the cumulative interaction scores for each gene were calculated. Genes were ranked according to the cumulative interaction scores. The dashed line separates the 5% top genes with highest cumulative interaction score with other genes. **(B)** Permutation plot of number of overlapped genes between the randomly sampled background genes and the 5% top ranked genes. Yellow and blue line indicates the overlap observed between the up- (Left) or down-regulated (Right) genes and the 5% top ranked genes. **(C)** Violin plot shows distribution of cumulative interaction score of each gene. **P* < 0.05, Dunn's test. **(D-F)** Compared to downregulated genes, significant interactions related to upregulated genes exhibited higher number **(D)**, higher intensity **(E)** and longer distance **(F)**. **(G)** Downregulated genes showed more proportion of promoter-associated contacts than upregulated genes. **(H)** Chromatin states were categorized into seven groups by ChromHMM, using the published ChIP-seq data encompassing various histone modifications including H3K27ac (GSE63137), H3K4me1 (GSE63137), H3K4me3 (GSE63137), H3K27me3(GSE63137), H3K9me3(GSE189755) and CTCF (GSE231067), along with ATAC-seq data (GSE150533). The enrichment for the categories is shown. The red box highlights the most significant enrichment of categories in the chromatin state. Specifically, up-regulated genes and their corresponding anchors were mainly enriched in the enhancer region (1, EnhLo; 2, Enh). Meanwhile, down-regulated genes and their corresponding anchors were primarily enriched in gene promoter regions (3, Tss). In addition, gene anchors were also enriched in CTCF regions (4, CTCF). **(I)** Representative map tracks show chromatin contacts and enhancer marks (H3K27ac, H3K4me1) associated with Up_genes- (Left) and Down_genes (Right).
