# Supplementary material for: Distinct epigenetic modulation of differentially expressed genes in the adult mouse brain following prenatal exposure to low-dose bisphenol A
Source: Cell Biol Toxicol. 2024 May 22;40(1):37. doi: 10.1007/s10565-024-09875-4 (PMC11111541; doi:10.1007/s10565-024-09875-4)
Supplement: Supplementary file 2 — Supplementary file2 (DOCX 18.0 KB) [file 10565_2024_9875_MOESM2_ESM.docx]

**Supplemental Methods**

**Title:** Distinct epigenetic modulation of differentially expressed genes in the adult mouse brain following prenatal exposure to low-dose bisphenol A

**Journal:** Cell Biology and Toxicology

**Authors:** Jie Weng^1†^, Yue-yan Zhu^1†^, Li-yong Liao^1^, Xin-tong Yang^2^, Yu-hao Dong^1^, Wei-da Meng^3^, Dai-jing Sun^1^, Yun Liu^3^, Wen-zhu Peng^1*^, Yan Jiang^1*^

*^1^Institutes of Brain Science, State Key Laboratory of Medical Neurobiology and MOE Frontiers Center for Brain Science, Fudan University, 200032, Shanghai, China.*

*^2^Shanghai Medical college, Fudan University, 200032, Shanghai, China.*

*^3^The MOE Key Laboratory of Metabolism and Molecular Medicine, Department of Biochemistry and Molecular Biology, School of Basic Medical Sciences, Fudan University, 200032, Shanghai, China.*

^†^JW and YZ contributed equally to this work

^*^WP and YJ are co-senior authors

**Correspondence**: Yan Jiang, [Yan_jiang@fudan.edu.cn](mailto:Yan_jiang@fudan.edu.cn)

***Open field test***

A 40cm×40cm×40cm open field box with white inner wall was used in this test. Mice were individually introduced to the open field box and allowed free exploration for 15 min. The whole procedure was video-recorded and analyzed by EthoVision XT 14.0. The virtual center zone was defined as half of the whole arena right in the middle of the bottom. Total distance was calculated to assess the mouse activity and time in center zone was analyzed to evaluate anxiety-like behavior.

***Elevated plus maze test***

A “+” shaped maze with two opposite positioned open arms and two opposite positioned closed arms were used. Mice were individually introduced to the elevated plus maze and allowed free exploration for 5 min. The whole procedure was video-recorded and analyzed by EthoVision XT 14.0. Total distance was calculated to assess the mouse activity and the preference of being in the open arms over closed arms was analyzed to assess the anxiety-like behavior.

***Forced swim test***

Fill a glass beaker with 30 cm-deep water at 25±1°C. Mice were individually introduced to the glass baker and record with a camera for 5 min. EthoVision XT 14.0 was used for analyzing the mobility. Immobility time were calculated to evaluate the depressive-like behavior.

***Tail suspension test***

The mice were secured with tape at the posterior 1/3 of the tail and hung head down on a stand, recorded by a camera for 5 min. EthoVision XT 14.0 was used to analysis the mobility. The duration of immobility was calculated to reflect the depressive-like behaviors.

***Integrative analysis of bulk RNA-seq and scRNA-seq***

The cell-type enrichment analysis was performed as described in the Methods session of main manuscript. A different set of published scRNA-seq (GSE211099) data of adult mouse motor cortex (WT-PBS group) was used.

***Hi-C analysis***

All analysis were performed as described in the Methods session of main manuscript. A different batch of published data from GSE168524 (Hi-C), GSE63137 (H3K27ac, H3K4me1, H3K4me3, H3K27me3), GSE189755 (H3K9me3), GSE231067 (CTCF) and GSE150533 (ATAC-seq) was used.
